# Meta-analysis of neural correlates of working memory, reward, and emotion processing in major depressive disorder using AES-SDM

**DOI:** 10.1186/s12888-026-08154-2

**Published:** 2026-05-19

**Authors:** Hui Ding, Ying Lu, Yongzhe Hou, Xin Chen, Ji Peng, Xizhao Chen, Jing Li, Qin Zhang

**Affiliations:** 1 Department of Radiology, The Second People’s Hospital of Guizhou Province, Guizhou Guiyang, 550004 China; 2 School of Basic Medical Sciences, Guizhou University of Traditional Chinese Medicine, Guizhou Guiyang, 550025 China; 3 Department of Women’s and Children’s Psychiatry, Guizhou Second People’s Hospital, Guizhou Guiyang, 550004 China

**Keywords:** Major depressive disorder, Working memory, Emotion, Reward processing, fMRI, AES-SDM meta-analysis

## Abstract

**Objective:**

We conducted coordinate-based anisotropy effect size signed differential mapping (AES-SDM) meta-analyses to identify task-general and domain-specific (working memory, reward, and emotion processing) activation abnormalities in patients with major depressive disorder (MDD).

**Materials and methods:**

We systematically searched the PubMed, Embase, Web of Science, ScienceDirect, and China National Knowledge Infrastructure databases for functional magnetic resonance imaging (fMRI) studies that compared patients with MDD with healthy controls (HCs) up to December 3, 2024. Differences in brain activity were evaluated using AES-SDM software across all task types, including emotion-processing, working memory, and reward-processing domains, to compare MDD patients with HCs.

**Results:**

Forty-six studies (11 on working memory, 12 on reward processing, and 23 on emotion processing) involving 1,558 patients with MDD and 1,468 HCs were included. Across all task types, patients with MDD showed greater activation in the left lenticular nucleus/putamen, right rolandic operculum, left anterior cingulate/paracingulate gyri, and right inferior frontal gyrus. In the emotion-processing domains, MDD was associated with hyperactivation in the right amygdala and left striatum. No clusters survived the primary corrected threshold in the working memory or reward-processing domains. Jackknife analyses supported the robustness of the main clusters, and Egger’s test did not indicate significant publication bias (all *p* > 0.05). Meta-regression did not reveal significant effects of age or illness duration on the results.

**Conclusions:**

This meta-analysis demonstrated task-general hyperactivation across salience/control-related regions in patients with MDD and domain-specific hyperactivation in the amygdala–striatal circuitry during emotion processing. The absence of corrected findings in working memory and reward processing highlights the need for more standardized paradigms and larger datasets to clarify domain-specific abnormalities.

**Clinical trial number:**

Not applicable.

**Supplementary Information:**

The online version contains supplementary material available at 10.1186/s12888-026-08154-2.

## Background

Major depressive disorder (MDD) is among the most prevalent and disabling psychiatric conditions. It is characterized by persistent low mood, anhedonia, and suicidal ideation, which significantly impair patients’ social functioning and quality of life. This disorder often recurs, contributing to increased morbidity and mortality rates [1]. Although the prevalence of MDD varies across countries and regions [2], its global incidence continues to increase annually, highlighting the urgent need for improved understanding and intervention [3]. Furthermore, owing to the substantial heterogeneity of psychiatric symptoms and the lack of objective diagnostic biomarkers, individualized and precise treatment for specific MDD subtypes or individuals remains a major challenge [4]. Therefore, identifying the neural correlates of working memory, reward, and emotional processing prior to treatment is highly important.

A growing body of functional magnetic resonance imaging (fMRI) data has revealed abnormal brain activity in patients with MDD during key neurocognitive processes such as emotional regulation [5], reward processing [6], and working memory [7], suggesting widespread impairments in both affective and cognitive domains. In working memory tasks, particularly n-back paradigms, patients with MDD frequently exhibit dysfunction in the prefrontal cortex, particularly the dorsolateral prefrontal cortex (dlPFC), with findings ranging from hypoactivation, indicating deficits in cognitive control, to hyperactivation, which possibly reflects compensatory mechanisms [8]. Pharmacological interventions such as vortioxetine have been shown to modulate activity in the right dlPFC and left hippocampus, suggesting a potential role in restoring cognitive network function [9]. In the reward-processing domain, patients with MDD demonstrate heterogeneous patterns of neural activation across different reward stages. Some studies have reported hyperactivation in regions such as the anterior cingulate cortex, middle frontal gyrus, cerebellum, and nucleus accumbens during reward anticipation or reception [10], whereas others have observed hypoactivation in areas such as the superior frontal gyrus during expected loss or reward feedback processing, indicating impairments in reinforced learning and affective regulation [11]. Meta-analyses have further suggested that, in addition to classical reward-related regions such as the striatum and anterior cingulate cortex, the insula, posterior cingulate cortex, and supplementary motor area are involved, reflecting widespread abnormalities in the reward-processing network [12, 13]. Notably, blunted activation in the anterior cingulate cortex and subgenual cortex during reward anticipation has been reported, which may underlie motivational deficits and anhedonia in MDD [14]. With respect to emotional processing, patients with MDD consistently exhibit exaggerated responses to negative emotional stimuli. Regions such as the parahippocampal gyrus, insula, and caudate nucleus show heightened activation when negative facial expressions or affective images are processed, reflecting disrupted emotion-related memory and regulatory mechanisms [15–17]. Similar hyperactivation patterns have been observed in the amygdala, middle temporal gyrus, and superior temporal gyrus, suggesting increased emotional sensitivity and biased negative appraisal [18]. Conversely, diminished responses to positive stimuli have been reported in areas such as the anterior cingulate cortex, lateral occipital cortex, and fusiform gyrus [19, 20], indicating impaired positive emotion processing. Furthermore, during emotional conflict tasks, patients with MDD exhibit decreased behavioral accuracy and reduced activation in brain regions associated with facial recognition and emotional evaluation, supporting the notion of impaired emotional regulation and emotion–cognition integration.

Although an increasing number of fMRI studies have reported abnormal neural activity during emotional processing, reward processing, and working memory in patients with MDD, the findings remain highly inconsistent across studies. Such discrepancies may be driven by substantial heterogeneity in sample characteristics (e.g., symptom severity, illness course, and medication status), task paradigms (e.g., cognitive load, reward stage, and stimulus valence), and imaging/analysis parameters. This variability not only underscores the complexity of MDD pathophysiology but also limits the interpretability and reproducibility of individual reports, highlighting the need for quantitative synthesis using standardized and transparent meta-analytic procedures. In addition, while previous meta-analyses have summarized aberrant neural responses within specific functional domains of MDD [12, 13], the existing evidence remains largely fragmented because most prior work has focused on a single domain or a particular task component in isolation. Consequently, it remains unclear whether reported abnormalities primarily reflect domain-specific dysfunction (e.g., emotion-related circuitry) or, more general, task-related neural alterations that are shared across cognitive–affective domains.

To address these gaps, the present study applied anisotropic effect-size signed differential mapping (AES-SDM) in a unified whole-brain, coordinate-based meta-analytic framework to examine task-based fMRI differences between adults with MDD and healthy controls (HCs). AES-SDM is a validated coordinate-based meta-analytic approach that reconstructs voxel-wise effect-size maps from reported peak coordinates and corresponding statistics, enabling quantitative whole-brain synthesis while accounting for spatial uncertainty and between-study variability [21].

Notably, rather than synthesizing each domain in separate meta-analyses with potentially different inclusion criteria and analytic decisions, we simultaneously conducted (i) a task-general meta-analysis pooling all eligible task types and (ii) parallel domain-specific meta-analyses for three major task domains—emotion processing, working memory, and reward processing—within the same analytical design. By integrating task-general and domain-specific perspectives in a single study, this approach enables a more direct comparison of shared versus domain-dependent neural abnormalities, clarifies whether neural alterations in MDD converge across tasks or are selectively expressed in particular domains, and aims to identify more robust and reproducible patterns of task-evoked brain activation abnormalities in MDD.

## Materials and methods

### Literature search

This review was registered with PROSPERO (ID: CRD420251101693). A systematic search was conducted for relevant studies in the PubMed, Embase, Web of Science, ScienceDirect and CNKI databases up to December 3, 2024. We used the following key words (using both free-text and MeSH search): ((“fMRI” [tw] OR “fmri”[tw] OR fmr imag* [tw] OR functional magnetic* [tw] OR “functional magnetic resonance imaging” [tw] OR “functional mri” [tw] OR “functional mr” [tw]) OR (“Magnetic Resonance Imaging”[mesh] AND “functional” [tw]) OR ((“Magnetic Resonance Imaging”[mesh] OR MR imag* [tw] OR “MRI”[tw] OR “magnetic resonance”[tw]) AND (“Functional Neuroimaging”[Mesh: noexp] OR functional imag*[tw] OR functional neuroimag* [tw]))) AND (“depression” [Mesh] OR “Depressive Disorder”[Mesh] OR “depression”[tw] OR “Depressive Disorder”[tw] OR “major depressive disorder”[tw] OR “major depression”[tw] OR “depressive”[tw] OR “depressezd” [tw] OR “depression neurosis” [tw] OR “melancholia” [tw] OR Depressive disorder* [tw] OR depress* [tw] OR depression disease* [tw] OR depressive disease* [tw] OR “Depression Disease” [tw] OR depressive Disease* [tw]) NOT (“Animals” [mesh] NOT “Humans” [mesh]).

Manual screening of all potentially relevant results was also conducted. Studies were considered eligible on the basis of the following criteria: (1) task-based fMRI studies involving individuals with MDD; (2) reported whole-brain analysis results in a standard stereotaxic space (Montreal Neurological Institute [MNI] or Talairach coordinates); (3) included participants aged 18 years or older; (4) incorporated a task assessing at least one of the following domains: working memory, reward processing, or emotion processing; and (5) compared adults with MDD and HCs.

The exclusion criteria were as follows: (1) conditions that might influence brain function or any contraindications for MRI scanning; (2) literature reviews, systematic reviews, meta-analyses, methodological papers, case reports, letters, conference abstracts, and editorials; (3) studies employing only regions of interest (ROIs) or seed voxel-based analyses; and (4) studies that did not report activation coordinates or associated statistical values (e.g., T values).

Two physicians independently conducted the literature search, and any disagreements were resolved through discussion to reach consensus. Finally, the included studies were categorized into one or more of the three domains. Study selection was performed in accordance with the PRISMA guidelines.

### AES-SDM analysis

AES-SDM software was used to analyze differences between patients with MDD and HCs across all task types, including the emotion processing, working memory, and reward-processing domains [22]. Peak coordinates reported in Talairach space were converted to MNI space by using an online tool (http://www.brainmap.org/icbm-2tal/). When the results were presented as z values, they were converted to t values for analysis (www.sdmproject.com/utilities/?show=Statistics). AES-SDM reconstructed effect size and statistical parameter maps reflecting increased and decreased brain region activation across individual studies. The Monte Carlo random-effects model implemented in AES-SDM integrated these statistical maps, with the significance threshold set at FWHM = 20 mm, 1000 permutations, TFCE-corrected *p* < 0.05, and a cluster extent threshold ≥ 50 voxels. Cluster coordinate reconstruction involved converting peak t-values to Hedges’ g, followed by the application of a Gaussian kernel to perform nonuniform smoothing of adjacent peak coordinate voxels. Volume rendering of cortical clusters showing significant differences was conducted in MNI standard space using MRIcron software.

Egger’s regression test, integrated within the AES-SDM software, was used to evaluate publication bias on the basis of effect sizes of peak voxels within significant clusters. Heterogeneity among studies was calculated to identify brain regions contributing to between-study variance. Jackknife sensitivity analysis was performed to assess the robustness of the results by iteratively repeating the meta-analysis, each time excluding one study across 11 working memory studies, 12 reward-processing studies, and 23 emotion-processing studies. Additionally, meta-regression analysis was performed using SDM-PSI 6.21 to assess whether study-level covariates—including age, sex distribution, medication status, and depression severity—significantly contributed to the variability of the observed effects.

## Results

### Literature search results

**Fig. 1:**
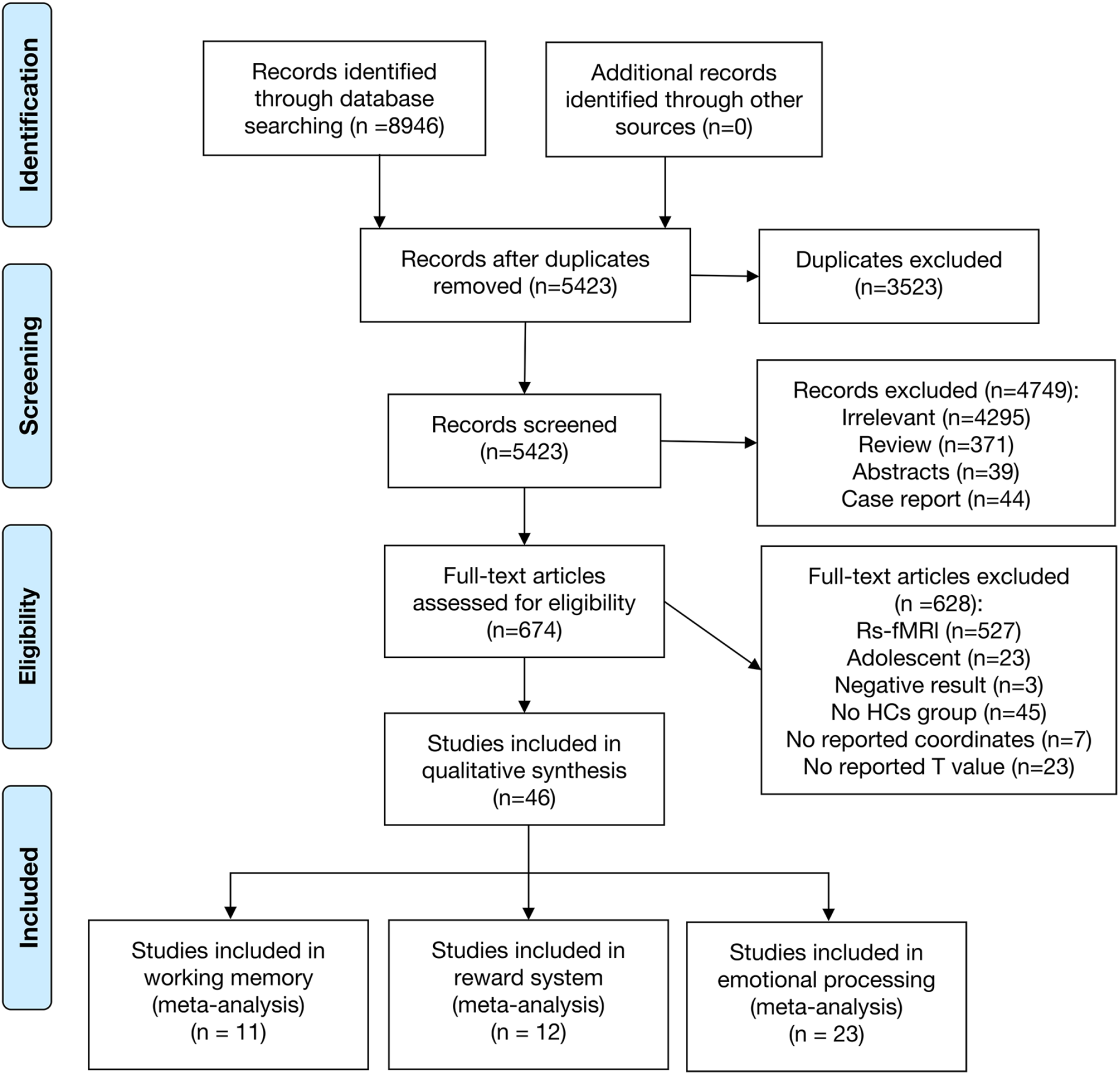
Flow chart of the study selection strategy

A total of 46 studies met the inclusion criteria and were included in the final meta-analysis [7–11, 14, 15, 17, 19, 20, 23–57]. These studies were categorized into three task domains (Fig. 1). Specifically, the working memory domain included 11 studies, comprising 380 patients with MDD and 343 HCs; the reward-processing domain included 12 studies, comprising 270 patients with MDD and 239 HCs; and the emotion-processing domain included 23 studies, comprising 908 patients with MDD and 886 HCs (Table 1).

**Table 1:** Descriptives of included fMRI studies of three domains of working memory, reward processing and emotion processing in the meta-analysis using a whole‐brain approach

	Sample size		Age (X ± S)		Medication	Depression Assessment Tools(MADRS/HAMD/BDI/IDS/HDRS/HRSD/DASS-21/CES-D /PHQ-9)and Scoring Results	Task	Corrective methods
Author, year	Patient (*n*) (female,%)	HCs (*n*) (female,%)	Patient	HCs	Unknowns (*n*) Antidepressants (*n*) Unmedicated (*n*)			
Working Memory Tasks								
Matsuo K, 2007 [7]	15, 67%	15, 60%	34.37 (11.5)	37.77 (12.1)	Unmedicated (8) Antidepressants (7)	HAMD 20.3 ± 5.3	n-back task	*p* < 0.05
Smith J, 2018 [9]	48, 56%	48, 54%	20–53	20–53	Antidepressants (48)	BDI-II 7.8 ± 6.7	n-back task	*p* < 0.05
Harvey PO, 2005 [23]	10, 42%	10,50%	19–45	18–42	Unknown (10)	MADRS 26.7 ± 4.6	n-back task	*p* <0.001
Vasic N,2009 [24]	14, 57%	14, 50%	37.0 (8.6)	32.6 (9.0)	Antidepressants (14)	MADRS 23.0 ± 4.0	a positive delay-related component	FDR *p* < 0.05
Lee TW,2013 [25]	14, 21%	14, 36%	65.1 (4.9)	64.8 (4.2)	Unknown (14)	HDRS 16.1 ± 5.8	one-back working memory task:	*p* < 0.05
Le TM, 2017 [26]	18, 67%	21, 57%	22.0 (3.09)	22.19 (3.38)	Unmedicated (18)	IDS 40.79 ± 8.70	delayed recognition task	FDR *p* < 0.05
Goodin P, 2019 [27]	14, 57%	13, 77%	29.79 (9.62)	30.62 (9.99)	Unknown (14)	DASS-21 28.62 ± 6.99	n-back task	TFCE *p* < 0.05
Cao W, 2021 [28]	40, 70%	47, 60%	19.98 (4.70)	20.98 (2.34)	Unknown (40)	CES-D 62.30 ± 9.35	n-back task	FWE *p* < 0.05
Ma M. 2021 [29]	37, 62%	54, 54%	25.89 (4.75)	23.94 (3.05)	Unmedicated (10) Antidepressants (27)	HAMD-17 24.35 ± 5.60	“number calculation working memory” task	FWE *p* < 0.05
Schwefel MK, 2023 [30]	110, 48%	55, 62%	36.4 (10.0)	30.5 (7.3)	Unknown (110)	BDI-II 27.3 ± 7.7	n-back paradigm with numerical stimuli	FWE *p* < 0.05
Hempel M, 2024 [31]	60, 43%	52, 44%	39.5 (10.6)	37.6 (10.8)	Antidepressants (60)	BDI-II 31.8 ± 8.5	using the emotion back-task	GRF *p* < 0.05
Reward Tasks								
Dichter GS, 2012 [10]	19, 79%	19, 63%	24.5 ± 5.4	27.9 ± 6.3	Unmedicated (14) Antipsychotics (5)	BDI-II 2.6 ± 4.9	monetary incentive delay task	*p* < 0.05
Smoski MJ, 2011 [11]	9 (-)	13 (-)	34.4 ± 15.0	26.2 ± 6.3	Unmedicated (5) Antidepressants (4)	BDI 16.7 ± 4.9	monetary incentive delay task	*p* < 0.05
Smoski MJ,2009 [14]	14, 50%	15, 60%	34.8 (14.3)	30.8 ± 9.7	Unknown (40)	HAMD 18.5–28.5	wheel of fortune task	*p* < 0.005
Schiller CE, 2013 [32]	19, 79%	19, 63%	23.6 (4.1)	27.9 (6.3)	Unknown (19)	BDI-II 2.6 ± 4.9	monetary incentive delay task	*p* < 0.05
Dillon DG,2014 [33]	21, 52%	21, 43%	34.33 (12.16)	36.62 (13.32)	Unmedicated (21)	BDI-II 23.62 ± 7.17	reward tokens	FWE *p* < 0.05
Hall GB,2014 [34]	15, 73%	15, 67%	47.67 (9.53)	46.33 (11.36)	Unknown (15)	BDI 26.29 ± 11.17 HRSD 17.93 ± 7.03	money game	FDR *p* < 0.05
Ubl B, 2015 [35]	23, 69%	23, 61%	41.17 (12.08)	42.74 (12.19)	Unmedicated (23)	BDI-II 5.08 ± 4.15	reward paradigm	*p* < 0.05
Takamura M, 2017 [36]	18, 67%	18, 67%	44(13.2)	38.3(8.46)	Antidepressants (18)	BDI-II 30.8 ± 11.29	monetary incentive delay task	FWE *p* < 0.05
Martin-Soelch C,2020 [37]	16, 75%	16, 75%	24.31 (4.08)	25.19 (4.79)	Unknown (16)	BDI-II 31.38 ± 10.94	monetary incentive delay task	*p* < 0.005
Rupprechter S,2021 [38]	18, 83%	16, 63%	18–33	17–41	Unknown (18)	HAMD 18.8 ± 6.9	a novel pavlovian conditioning task	*p* < 0.005
Mielacher C, 2024 [39]	53, 51%	41, 54%	41.58 (13.09)	40.61 (13.22)	Antidepressants (53)	BDI-II 33.34 ± 8.75	reward paradigm	*p* < 0.05
Taylor CT, 2024 [40]	45, 68.9%	23, 69.6%	30.2 (9.8)	29.7 (8.4)	Unknown (45)	PHQ-9 13.0 ± 4.4	social incentive delay task	*p* < 0.05
Emotion Tasks								
Kerestes P,2012 [8]	19, 78%	20, 50%	33.6 (13.64)	35.8 (12.10)	Unmedicated (19)	HAMD 1.79 ± 1.27	emotional face gender-labeling task	FWE *p* < 0.05
Tak S, 2021 [15]	34, 100%	28, 100%	24.5(2.8)	24.4(2.6)	Unmedicated (34)	BDI 33.7 ± 7.1	negative emotional task	*p* < 0.005
Lemke H,2022 [17]	333,65%	333,60%	36.80 (12.57)	36.64 (12.49)	Unknown (333)	HDRS 19.61 ± 5.88	negative emotional face-processing paradigm	FWE *p* < 0.05
Zhang S,2022 [19]	42, 61.9%	32, 78.1%	22.19 (1.97)	21.50 (3.13)	Unknown (42)	HAMD-24 15.67 ± 3.56 BDI 24.10 ± 7.43	fMRI stimuli and task	FWE *p* < 0.05
Alders GL,2019 [20]	48, 69%	30, 30%	34.7(12.2)	33.2(9.8)	Antidepressants (48)	MADRS 30 ± 6	emotional conflict task	*p* < 0.05
Beauregard M, 2006 [41]	12, 75%	12, 75%	43 (11)	45 (10)	Unknown (12)	HDRS 25 ± 6	sad affect face recognition task	*p* < 0.05
Aizenstein HJ ,2011 [42]	33, 64%	27, 70.3%	67.7 (5.2)	71.6 (7.5)	Antidepressants (33)	HAMD 19.7 ± 3.7	faces and shapes affective reactivity task	*p* < 0.005
Mannie ZN, 2011 [43]	28, 57%	28, 46%	18.79 (1.40)	19.68 (1.44)	Unknown (28)	BDI 4.21 ± 3.75	simple perceptual task	*p* < 0.05
Ritchey M, 2011 [44]	22, 59.1%	14, 64.3%	36.1 (10.1)	34.6 (6.9)	Unmedicated (22)	BDI 25.1 ± 8.8	emotion evaluation task	*p* < 0.05
Wang Y,2012 [45]	18, 61%	18, 61%	31.61(7.9)	31.67(6.8)	Unknown (18)	HAMD 19.9 ± 5.3	emotional pictures	*p* < 0.005
Aust S,2013 [46]	14, (64%)	14, (57%)	55.1(11.3)	54.9(11.8)	Unknown (14)	HDRS>8 MADRS >11 BDI 13	music and human emotional faces	FDR *p* < 0.05
Chechko N,2013 [47]	18, 72%	18, 72%	36.5 ( 10.8)	36.0 (10.3)	Unknown (18)	BDI 31.7 ± 8.2	emotional interference task	FWE *p* < 0.05
Feeser M,2013 [48]	19, 32%	19, 32%	43.9 (8.7)	38.2 (11.9)	Unmedicated (19)	HRSD 20.3 ± 4.3 BDI 27.9 ± 9.6	anticipatory emotional processes	alpha *p* < 0.05
Furey ML, 2013 [49]	15, 27%	21, 43%	32.9(7.8)	30.5(8.5)	Unmedicated (15)	MADRS 30 ± 6.0	face-identity and face-emotion working memory tasks	alpha *p* < 0.05
Korgaonkar MS, 2013[50]	30, 60%	30, 60%	41.2 (15.8)	35.7 (14.1)	Unknown (30)	HRSD 19.2 ± 3.1	emotion task	*p* < 0.005
Victor TA, 2013 [51]	10, 60%	10,70%	33.2 (5.0)	28.4 (5.7)	Antidepressants (10)	HAMD 24.8 ± 5.8	back- ward masking task	FWE *p* < 0.05
Miskowiak KW, 2015 [52]	13, 62%	17, 65%	48.6 (14.7)	42.5 (10.2)	Unknown (13)	BDI 1.4 ± 2.1	faces dot-probe task	FWE *p* < 0.05
Koch K,2018 [18]	30, 53%	30, 47%	29.9(8.9)	26.8(4.3)	Unknown (30)	BDI 29.5 ± 8.3 HRSD 20.9 ± 5.1	emotion task	FWE *p* < 0.05
Huang CM, 2019 [53]	40, 63%	55, 69%	68.10(5.3)	66.36(5.42)	Antidepressants (40)	HAMD 11.66 ± 6.41	color–word stroop task	FWE *p* < 0.05
Keller M,2021 [54]	39, 44%	37, 41%	35.2(13.6)	32.3(12.8)	Antidepressants (39)	HAMD 16.5 ± 7.5 BDI 26.8 ± 11.5	NF task	FDR *p* < 0.05
Fang Z,2022 [55]	18, 78%	20, 75%	32.11(10.8)	29.5(8.6)	Unmedicated (18)	BDI 5.15 ± 5.52	emotional stroop task	FWE *p* < 0.05
Zhang Q,2022 [56]	36, 56%	36, 58%	34.32 (5.45)	32.38 (9.24)	Unmedicated (36)	HDRS 23.6 ± 3.64	emotional subregion	FDR *p* < 0.05
Klug M, 2024 [57]	37, 54%	37, 43%	38.43 (12.37)	32.95(10.67)	Unknown (37)	HDRS 23.08 ± 4.87	subliminal negative emotion processing	FWE *p* < 0.05

HCs: Healthy Controls; MADRS: Montgomery–Asberg Depression Rating Scale; HAMD: Hamilton Depression Rating Scale; HDRS: Hamilton Depression Rating Scale; HRSD: Hamilton Rating Scale for Depression; BDI: Beck Depression Inventory; IDS: Inventory of Depressive Symptomatology; DASS-21: Depression Anxiety Stress Scales-21; CES-D: Center for Epidemiologic Studies Depression Scale; PHQ-9: Patient Health Questionnaire-9

In terms of paradigm distribution, n-back-related paradigms were the most frequently represented in the working memory domain, whereas monetary incentive delay paradigms were the most frequently represented in the reward-processing domain. By contrast, the emotion-processing domain was more heterogeneous, with no single paradigm clearly predominating across studies; the included studies covered a range of face-based, negative emotion-related, and other affective paradigms.

### Meta-analysis results

#### Meta-analyses across all task types

**Fig. 2:**
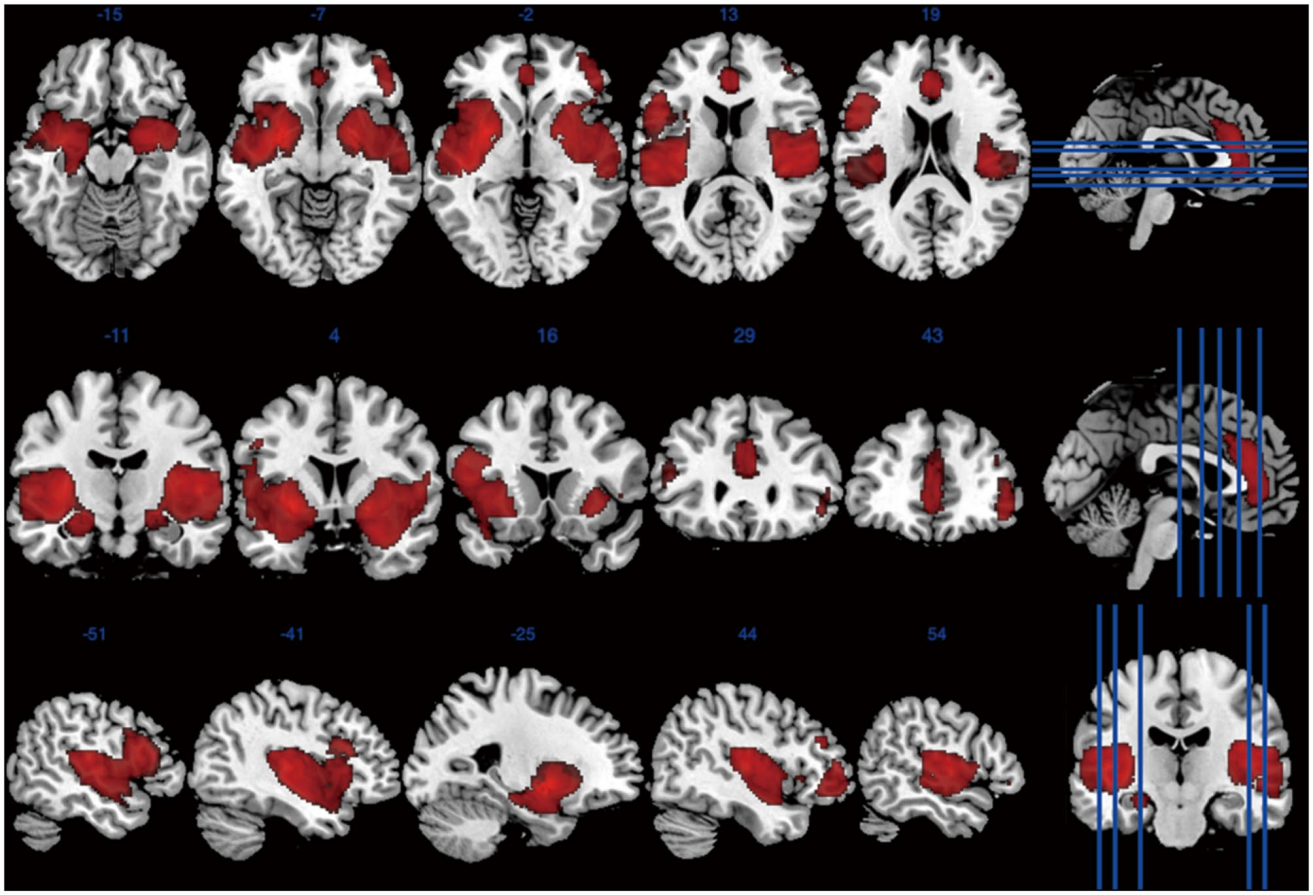
Abnormal brain regions identified through SDM meta-analysis across all task types in neuroimaging studies. Regions with significantly increased activation in patients with MDD relative to HCs are highlighted in red

Compared with HCs, individuals with MDD exhibited hyperactivation in the left lenticular nucleus/putamen, right rolandic operculum, left anterior cingulate/paracingulate gyri, and right inferior frontal gyrus across all task types (Fig. 2). Egger’s test revealed no significant publication bias for the left lenticular nucleus/putamen (Bias = 0.21, Z = 0.51, *p* = 0.613), right rolandic operculum (Bias = 0.71, Z = 1.14, *p* = 0.252), left anterior cingulate/paracingulate gyri (Bias = 0.64, Z = 1.29, *p* = 0.198), or right inferior frontal gyrus (Bias = 0.71, Z = 1.14, *p* = 0.252), indicating low between-study heterogeneity (Table 2). However, no significant hypoactivation was observed across all the task types.

**Table 2:** Applying the SDM method to study brain functional changes across all task types in MDD

Research methods	Anatomical label	Peak MNI coordinate			SDM-Z	*p* value	Voxels	Jackknife	Egger
		X	Y	Z				sensitivity analysis	Bias test
All task types									
MDD > HCs									
	Left lenticular nucleus / putamen, BA 48	-28	2	-2	4.379	0.01000009	7241	43 / 46	Bias:0.21 Z:0.51 p:0.613
	Right rolandic operculum, BA 48	52	-10	10	3.971	0.000999987	5962	44 / 46	Bias:0.71 Z:1.14 p:0.252
	Left anterior cingulate / paracingulate gyri, BA 24	-2	36	20	3.699	0.004999995	981	43 / 46	Bias:0.64 Z:1.29 p:0.198
	Right inferior frontal gyrus, BA 45	44	36	26	2.774	0.042999983	60	42 / 46	Bias:0.71 Z:1.14 p:0.252
HCs > MDD	—	—	—	—	—	—	—	—	—

HCs: Healthy Controls; MDD: Major Depressive Disorder; MNI: Montreal Neurological Institute; BA: Brodmann Area

#### Meta-analyses across working memory, reward processing and emotion processing

**Fig. 3:**
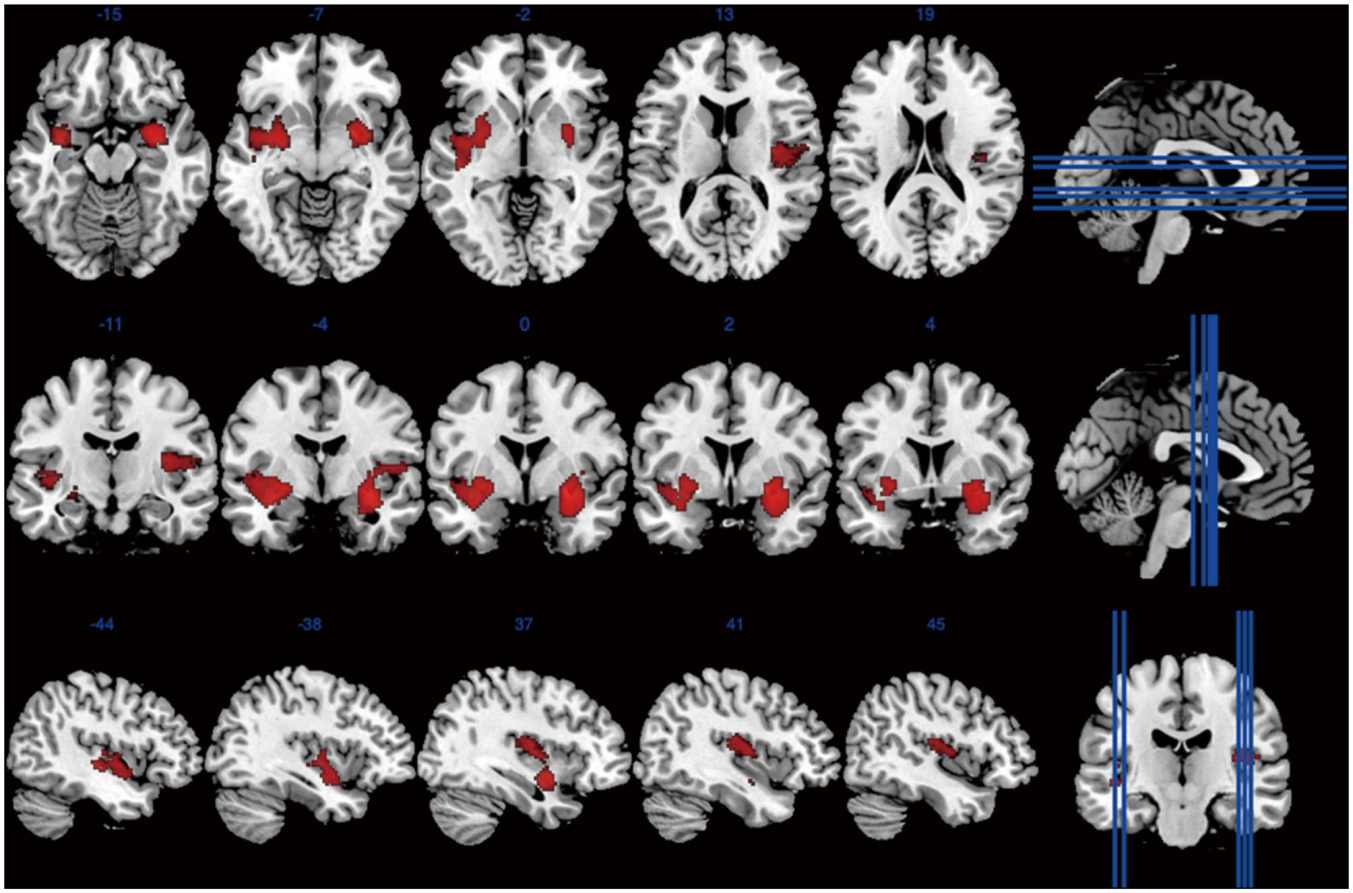
Abnormal brain regions identified through SDM meta-analysis across emotion processing domains in neuroimaging studies. Regions with significantly increased activation in patients with MDD relative to HCs are highlighted in red

Compared with HCs, individuals with MDD exhibited hyperactivation in the right amygdala and left striatum during emotion processing (Fig. 3). Egger’s test did not indicate significant publication bias for the right amygdala (Bias = 0.09, Z = 0.16, *p* = 0.870) or the left striatum (Bias = 0.08, Z = 0.15, *p* = 0.880), suggesting low heterogeneity among the included studies (Table 3). However, no significant hypoactivation was observed in this domain. Additionally, neither hyperactivation nor hypoactivation was identified in the working memory or reward-processing domains.

**Table 3:** Applying the SDM method to investigate brain functional alterations across emotion processing domains in MDD

Research methods	Anatomical label	Peak MNI coordinate			SDM-Z	*p* value	Voxels	Jackknife	Egger
		X	Y	Z				sensitivity analysis	Bias test
Emotion processing domain									
MDD > HCs									
	Right amygdala, BA 34	32	-2	-18	4.032	0.003000021	866	20/23	Bias:0.09 Z:0.16 p:0.870
	Left striatum	-26	-4	-6	3.546	0.009000003	770	21/23	Bias:0.08 Z:0.15 p:0.880
HCs > MDD	—	—	—	**—**	**—**	—	—	—	—
Working memory domain	—	—	—	**—**	**—**	—	—	—	—
Reward processing domain	—	—	—	**—**	**—**	—	—	—	—

HCs: Healthy Controls; MDD: Major Depressive Disorder; MNI: Montreal Neurological Institute; BA: Brodmann Area

#### Exploratory analyses

In exploratory analyses using a more lenient uncorrected threshold (*p* < 0.05, k ≥ 50 voxels), several subthreshold clusters were identified in both the working memory and reward-processing domains (Supplementary Tables S1–S2). However, none of these clusters survived the primary correction threshold applied in the main analyses.

### Jackknife sensitivity and meta-regression analysis results

Jackknife sensitivity analysis demonstrated robust results, with findings for the left lenticular nucleus/putamen replicated in 43 of 46 iterations, the right rolandic operculum in 44 of 46 iterations, the left anterior cingulate/paracingulate gyri in 43 of 46 iterations, and the right inferior frontal gyrus in 42 of 46 iterations (Table 2). Findings for the right amygdala were replicated in 20 of 23 iterations and those for the left striatum were replicated in 21 of 23 iterations (Table 3). Meta-regression analysis revealed no significant impact of differences in age, sex, medication or severity of MDD between the groups.

## Discussion

This AES-SDM meta-analysis synthesized task-based fMRI evidence in adults with MDD and identified convergent patterns of task-evoked hyperactivation. Specifically, the emotion-processing domain showed hyperactivation in the right amygdala and left striatum. By contrast, no clusters survived the primary corrected threshold in the working memory or reward-processing domains. When all task types were pooled, MDD was associated with increased activation in the left lenticular nucleus/putamen, right rolandic operculum, left anterior cingulate/paracingulate gyri, and right inferior frontal gyrus.

### Task-general functional abnormalities across all task types in MDD

When all task types were considered together, compared with HCs, patients with MDD showed increased task-related activation in the left lenticular nucleus/putamen, right rolandic operculum, left anterior cingulate/paracingulate gyri, and right inferior frontal gyrus, whereas no consistent hypoactivation was observed. These findings suggest that cross-task neural abnormalities in MDD are not restricted to a single cognitive or affective dimension but may instead reflect a broader functional imbalance involving salience processing, cognitive control, response inhibition, and sensorimotor integration.

Specifically, the putamen, as a key component of the striatum, is closely involved in motivational drive, habitual response, and action selection [58]; the anterior cingulate/paracingulate cortex is implicated in conflict monitoring, emotion regulation, and cognitive control [59]; the inferior frontal gyrus plays important roles in inhibitory control and salience-oriented processing [60]; and the rolandic operculum may reflect abnormalities in sensorimotor integration and interoceptive-related processing [61]. The convergent hyperactivation across these regions may indicate that patients with MDD need to recruit greater amounts of neural resources to maintain task performance across different task demands, which is consistent with inefficient processing and/or compensatory engagement at the task-general level.

Overall, these results suggest that task-general abnormalities in MDD may be more prominently characterized by widespread hyperactivation within salience-, control-, and sensorimotor-related networks under current meta-analytic conditions.

### Significance of hyperactivation in brain regions involved in emotion processing in patients with MDD

Our meta-analysis revealed significant hyperactivation in the right amygdala and left striatum during emotion processing in patients with MDD, highlighting the pivotal role of these regions in the neuropathology of affective dysregulation. The amygdala is critically involved in the detection and appraisal of emotional stimuli [62], particularly those with negative valence, and its heightened activity has been consistently linked to increased emotional reactivity and biased processing of negative information in MDD [51, 54, 63]. This pattern may reflect heightened sensitivity to negative emotional cues, which is consistent with the clinical phenotype of persistent negative affect and increased reactivity to adverse stimuli observed in patients with MDD.

Similarly, the striatum, which includes the putamen and caudate nucleus, plays an essential role in reward-related processing and the motivational aspects of emotion [64, 65]. Hyperactivation in this region may reflect altered salience attribution and/or atypical engagement of reward-related learning mechanisms, which have been linked to anhedonia and altered affective experience [66]. Together, the observed amygdala and striatal hyperactivation may indicate dysregulated recruitment of subcortical affective–motivational circuitry during emotion processing in MDD [67].

Importantly, the present meta-analytic evidence is associative rather than causal. Nevertheless, the consistent involvement of the amygdala and striatum during emotion processing reinforces the view that dysregulated affective–motivational circuitry is a key neurobiological feature of MDD. From a translational perspective, these findings support the role of the amygdala–striatal circuit as a clinically relevant neural target that may help inform biomarker-based patient stratification and guide the development or optimization of circuit-focused treatments.

### Possible reasons for the lack of abnormal activation in working memory and reward processing in patients with MDD

The absence of significant hyperactivation or hypoactivation in the working memory and reward-processing domains in this meta-analysis may be attributed to several methodological and neurobiological factors. First, the heterogeneity in task paradigms across studies may have reduced the statistical power to detect convergent neural alterations [68]. Working memory tasks often differ in terms of cognitive load (e.g., 1-back vs. 3-back), modality (verbal vs. visuospatial), and response demands, leading to variability in the activation of frontoparietal and dorsolateral prefrontal regions commonly associated with executive control [69, 70]. Similarly, reward-processing studies encompass multiple phases (anticipation, outcome, or feedback) and involve diverse reward types (monetary, social, or primary), potentially engaging distinct neural circuits [71, 72]. This task variability could obscure consistent activation patterns in meta-analytic models, particularly when sample sizes within subdomains are modest.

Second, participant-related factors such as medication status, illness severity, comorbidities, and duration of illness may introduce interstudy variability. Qian’s study suggested that pharmacological interventions may alter neural responses during cognitive and reward tasks, particularly in frontostriatal circuits, potentially obscuring disorder-specific effects [73]. Moreover, reward deficits and executive dysfunction in MDD are often subtle and may be state dependent, emerging more clearly under conditions of acute symptom exacerbation or treatment resistance, which may not be fully captured in aggregated datasets.

Third, the use of stringent statistical thresholds and conservative correction methods (TFCE-corrected *p* < 0.05, extent ≥ 50 voxels) may have limited the detection of small but meaningful effects, especially in regions with lower signal-to-noise ratios or high interindividual anatomical variability. Additionally, the AES-SDM method relies on peak coordinate data rather than raw statistical images, which may lead to the underestimation of distributed or subthreshold activation differences in small sample subgroups.

### Interpretive boundaries and potential reasons for the absence of significant differences in activation in working memory and reward processing

In the present study, we did not detect statistically significant or consistently convergent whole-brain activation differences for working memory (11 studies) or reward processing (12 studies). Of note, this “null finding” should not be interpreted as evidence that working memory or reward-related processes are unaffected. A more plausible interpretation is that, given the currently available evidence base and the analytic framework and thresholding adopted in AES-SDM, we were unable to identify task-related activation abnormality patterns that were consistently reproducible across multiple studies and reached the statistical threshold.

From a methodological perspective, substantial heterogeneity existed across the studies. With respect to working memory, studies differed in task load (e.g., varying n-back levels), baseline/control and contrast definitions, and statistical strategies. With respect to reward processing, studies varied in terms of task phase (anticipation vs. outcome/feedback), reward type, and task structure. In addition, preprocessing pipelines, statistical thresholding, and covariate adjustment strategies were not uniform across studies. Such methodological variability may disperse the spatial distribution of reported peak coordinates, thereby reducing cross-study convergence and statistical detectability [74]. Moreover, clinical heterogeneity (e.g., depression severity, illness stage, and medication status) may contribute to inconsistencies in effect direction or spatial localization, further limiting the stability of meta-analytic results [75].

Although finer stratification by WM load/modality or reward phase/type may help reduce heterogeneity, the number of available studies within each subtype was too small and unevenly distributed to support reliable subgroup meta-analysis in the present dataset.

Overall, we interpret these findings cautiously as reflecting a lack of robust, cross-study, spatially consistent evidence. Future studies with more standardized paradigms and reporting, together with finer clinical stratification, are needed to further clarify the neural mechanisms of working memory and reward processing in MDD.

### Limitations

Several limitations of this meta-analysis should be acknowledged, which necessitate a cautious interpretation of the findings. First, the relatively small number of included studies may limit the robustness and generalizability of the results. Second, the use of peak coordinates extracted from published reports may not fully capture the spatial extent of alterations in brain activity, potentially introducing selection bias. Finally, because medication information was insufficiently reported in the primary studies—particularly given the presence of a substantial proportion of “unknown” cases—any medication-related conclusions should be interpreted with caution. Future studies should report medication details, including drug class, dosage, and treatment duration, more systematically.

## Conclusion

This meta-analysis revealed consistent hyperactivation in the right amygdala and left striatum during emotion-processing tasks in patients with MDD, but no significant abnormalities in working memory or reward-processing domains were identified. These findings emphasize the central role of heightened emotional reactivity in MDD, supporting the role of the amygdala and striatal circuits as potential neurobiological markers for emotional dysregulation. The lack of significant abnormalities in working memory and reward-related tasks may reflect differences in sample size, task paradigms, and imaging parameters.

Overall, our findings highlight the importance of targeting emotion-related neural circuits in future research and therapeutic interventions, while emphasizing the need for standardized task paradigms and larger, longitudinal samples to increase the translational relevance of neuroimaging biomarkers in clinical psychiatry.

## Electronic Supplementary Material

Below is the link to the electronic supplementary material.

Supplementary Material 1

## Data Availability

All data generated or analysed during this study are included in this published article and its supplementary information files. Further inquiries can be directed to the corresponding authors.

## Abbreviations

fMRI: Functional magnetic resonance imaging
AES-SDM: Anisotropic effect-size signed differential mapping
PRISMA: Preferred Reporting Items for Systematic Reviews and Meta-Analyses
PROSPERO: International Prospective Register of Systematic Reviews
WOS: Web of Science
CNKI: China National Knowledge Infrastructure
HCs: Healthy controls
MNI: Montreal Neurological Institute
ROI: Region of Interest
BA: Brodmann area
dlPFC: Dorsolateral prefrontal cortex
GRF: Gaussian random field
FDR: False discovery rate
FWE: Family-wise error
