# Supplementary material for: Meta-analysis of neural correlates of working memory, reward, and emotion processing in major depressive disorder using AES-SDM
Source: BMC Psychiatry. 2026 May 19;26:543. doi: 10.1186/s12888-026-08154-2 (PMC13390239; doi:10.1186/s12888-026-08154-2)
Supplement: Supplementary file 1 — Supplementary Material 1 [file 12888_2026_8154_MOESM1_ESM.docx]

Supplementary materials

**1. Supplementary Methods**

**1.1 Primary analysis threshold**

The primary coordinate-based meta-analyses were conducted using AES-SDM implemented in SDM-PSI. Statistical significance was determined using permutation-based correction as implemented in SDM-PSI. The predefined statistical threshold for the main analyses was set at:

TFCE-corrected

*p* < 0.05

1000 permutations

cluster extent ≥ 50 voxels

FWHM = 20 mm

Only clusters surviving this correction threshold were considered statistically significant in the primary analyses.

**1.2 Exploratory uncorrected analyses**

To further explore potential subthreshold activation patterns in the working memory and reward domains, additional AES-SDM analyses were conducted using a more lenient statistical threshold:

uncorrected *p* < 0.05

cluster extent ≥ 50 voxels

FWHM = 20 mm

No permutation-based or TFCE-based correction was applied in these exploratory analyses. These analyses were conducted post hoc and were considered hypothesis-generating.

**2. Supplementary Results**

**2.1 Working memory domain**

Using the exploratory uncorrected threshold (p < 0.05, k ≥ 50 voxels), several clusters showing greater activation in MDD relative to healthy controls were identified within canonical working memory–related regions. These included the left precentral gyrus, right middle frontal gyrus, left median cingulate/paracingulate gyri, right precuneus, right inferior parietal lobule (excluding supramarginal and angular gyri), left inferior parietal lobule (excluding supramarginal and angular gyri), right precentral gyrus, right middle occipital gyrus, left fusiform gyrus, right cerebellar hemispheric lobule VI, right inferior occipital gyrus, left inferior occipital gyrus, and left postcentral gyrus (see Table S1). No clusters were observed for the contrast HCs > MDD under this exploratory threshold.

**Table S1 Exploratory uncorrected AES-SDM results for the working memory domain (p < 0.05, k ≥ 50 voxels)**

| Research methods | Anatomical label | Peak MNI coordinate | | | SDM-Z | p value | Voxels |
| --- | --- | --- | --- | --- | --- | --- | --- |
|  |  | X Y Z | | |  |  |  |
| MDD > HCs |  | | | | | | |
|  | Left precentral gyrus, BA 44 | -50 | 6 | 22 | 2.888 | 0.001935720 | 1776 |
|  | Right middle frontal gyrus, BA 46 | 40 | 40 | 30 | 3.923 | 0.000043809 | 1312 |
|  | Left median cingulate / paracingulate gyri, BA 24 | -2 | 12 | 40 | 2.257 | 0.012018681 | 1410 |
|  | Right precuneus, BA 7 | 4 | -62 | 50 | 2.333 | 0.009813964 | 1032 |
|  | Right inferior parietal (excluding supramarginal and angular) gyri, BA 40 | 40 | -40 | 46 | 2.418 | 0.007808685 | 355 |
|  | Left inferior parietal (excluding supramarginal and angular) gyri, BA 40 | -38 | -44 | 46 | 2.738 | 0.003088593 | 313 |
|  | Right precentral gyrus, BA 6 | 36 | 2 | 50 | 2.342 | 0.009588420 | 287 |
|  | Right middle occipital gyrus, BA 18 | 32 | -84 | 8 | 2.304 | 0.010623574 | 262 |
|  | Left fusiform gyrus, BA 37 | -46 | -60 | -18 | 2.045 | 0.020423889 | 199 |
|  | Right cerebellum, hemispheric lobule VI, BA 37 | 26 | -58 | -26 | 1.898 | 0.028822184 | 156 |
|  | Right inferior occipital gyrus, BA 18 | 26 | -94 | -6 | 1.792 | 0.036554039 | 77 |
|  | Left inferior occipital gyrus, BA 19 | -38 | -86 | -6 | 1.868 | 0.030879617 | 56 |
|  | Left postcentral gyrus, BA 1 | -52 | -30 | 56 | 1.931 | 0.026718140 | 50 |
| HCs > MDD | —— | | | | | | |

**2.2 Reward domain**

Under the exploratory uncorrected threshold, several clusters with greater activation in MDD compared with healthy controls were observed in key reward-related regions, including: left lenticular nucleus (putamen), right striatum, left hippocampus, and right anterior thalamic projections (see Table S2). No clusters were detected for the contrast HCs > MDD under this threshold.

**Table S2 Exploratory uncorrected AES-SDM results for the reward domain (p < 0.05, k ≥ 50 voxels)**

| Research methods | Anatomical label | Peak MNI coordinate | | | SDM-Z | p value | Voxels |
| --- | --- | --- | --- | --- | --- | --- | --- |
|  |  | X Y Z | | |  |  |  |
| MDD > HCs |  | | | | | | |
|  | Left lenticular nucleus, putamen | -28 | -2 | 0 | 3.479 | 0.000252008 | 3609 |
|  | Right striatum | 20 | -2 | -6 | 3.863 | 0.000056088 | 3727 |
|  | Left hippocampus, BA 20 | -24 | -20 | -16 | 2.249 | 0.012264311 | 116 |
|  | Right anterior thalamic projections | 16 | 4 | 18 | 1.990 | 0.023270607 | 63 |
| HCs > MDD | —— | | | | | | |

1. **Supplementary Figures**


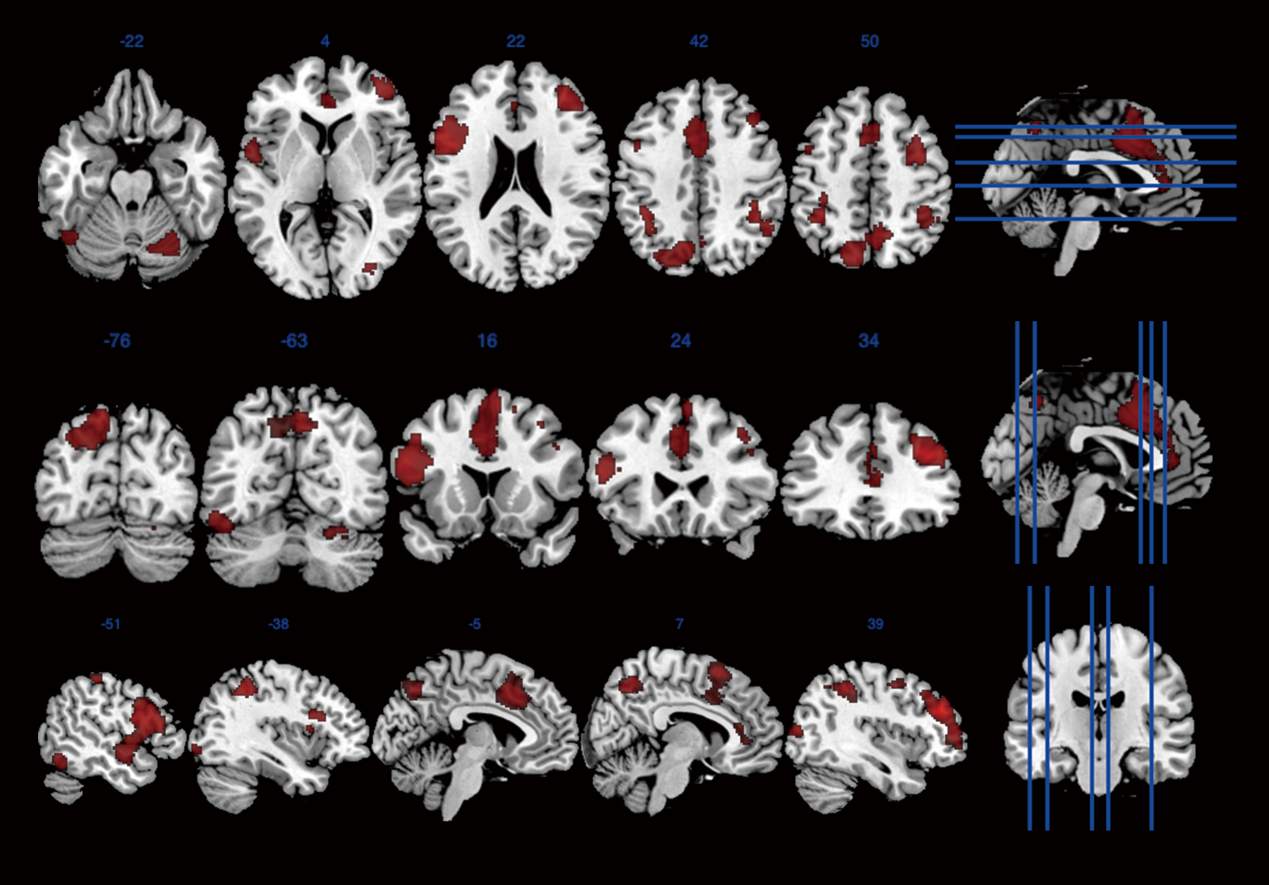


Figure S1 Exploratory uncorrected activation patterns for the working memory domain (MDD > HCs). Threshold: uncorrected p < 0.05, k ≥ 50 voxels.


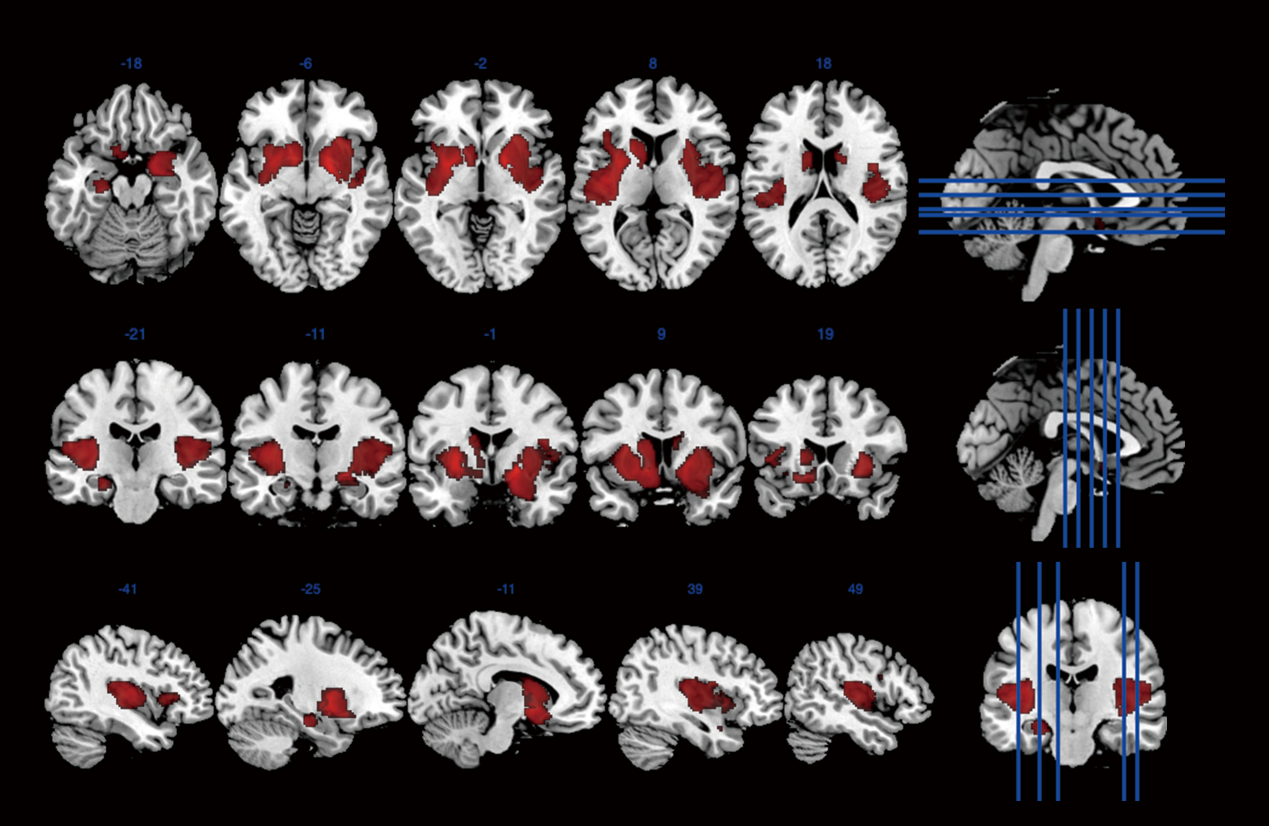


Figure S2 Exploratory uncorrected activation patterns for the reward domain (MDD > HCs). Threshold: uncorrected p < 0.05, k ≥ 50 voxels.

**4. Statistical caution statement**

These findings did not survive the predefined correction threshold used in the primary analyses and should therefore be interpreted as preliminary and hypothesis-generating rather than confirmatory.
